# A Self-Organized Model for Cell-Differentiation Based on Variations of Molecular Decay Rates

**DOI:** 10.1371/journal.pone.0036679

**Published:** 2012-05-31

**Authors:** Rudolf Hanel, Manfred Pöchacker, Manuel Schölling, Stefan Thurner

**Affiliations:** 1 Section for Science of Complex Systems/CeMSIIS, Medical University of Vienna, Vienna, Austria; 2 Santa Fe Institute, Santa Fe, New Mexico, United States of America; Semmelweis University, Hungary

## Abstract

Systemic properties of living cells are the result of molecular dynamics governed by so-called genetic regulatory networks (GRN). These networks capture all possible features of cells and are responsible for the immense levels of adaptation characteristic to living systems. At any point in time only small subsets of these networks are active. Any active subset of the GRN leads to the expression of particular sets of molecules (expression modes). The subsets of active networks change over time, leading to the observed complex dynamics of expression patterns. Understanding of these dynamics becomes increasingly important in systems biology and medicine. While the importance of transcription rates and catalytic interactions has been widely recognized in modeling genetic regulatory systems, the understanding of the role of degradation of biochemical agents (mRNA, protein) in regulatory dynamics remains limited. Recent experimental data suggests that there exists a functional relation between mRNA and protein decay rates and expression modes. In this paper we propose a model for the dynamics of successions of sequences of active subnetworks of the GRN. The model is able to reproduce key characteristics of molecular dynamics, including homeostasis, multi-stability, periodic dynamics, alternating activity, differentiability, and self-organized critical dynamics. Moreover the model allows to naturally understand the mechanism behind the relation between decay rates and expression modes. The model explains recent experimental observations that decay-rates (or turnovers) vary between differentiated tissue-classes at a general systemic level and highlights the role of intracellular decay rate control mechanisms in cell differentiation.

## Introduction

Understanding living cells at a systemic level is an increasingly important challenge in biology and medicine [1]–[5]. Regulatory interactions between intracellular molecular agents (e.g. DNA, RNA, proteins, hormones, trace elements), form so-called *genetic regulatory networks* (GRN), which orchestrate gene expression and replication, coordinate metabolic activity, and cellular development, respond to changes in the environment, or stress. GRN coordinate regulatory dynamics on all levels from cell-fate [6], [7] to stress response [8]–[10]. Qualitative understanding of GRN structure is for instance obtained from promoter sequences [11]–[13], gene-expression profiling [14]–[16] or protein-protein interactions (proteome) [17]. However qualitative information on GRN structure alone is insufficient to understand GRN dynamics. The structure of a GRN, i.e. its topology, is given by the way nodes in the network are connected by links. Nodes represent effector molecules (agents), as for instance genes, promoters, mRNA, siRNA, proteins, transcription factors, - and links represent either catalytic *up-* or *down*-regulation of the production of one agent by another agent. It has been recognized that quantitative information is required to understand the complex dynamical properties of regulatory interactions in living cells [18], [19], mainly because dynamics on interaction networks with identical topology still depends on the strength of interactions (links) between agents (nodes). Models of GRN dynamics aid the task of understanding properties of GRN at various levels of detail available in experimental data and therefore provide valuable tools for integrating information from different sources into unifying pictures and for reverse engineering GRN from experimental data. Any model should *adequately* reproduce GRN dynamics and *sufficiently* exhibit systemic properties of the GRN, including (i) homeostasis, (ii) multi-stability, (iii) periodic dynamics, (iv) alternating activity, (v) self-organized critical dynamics (SOC) and (vi) differentiability.

*Homeostatic dynamics* regulates the equilibrium concentration levels of agents, e.g. [20], *multi-stability* shows switching between multiple steady states [21], [22]. Examples for *periodic dynamics* are e.g. the cell-cycle [17], circadian-clock [23], I
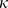
B-N
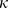
B signaling [24], hER dynamics [25], [26] etc. Some molecular agents show *alternating activity*, i.e. their concentrations alternate between being detectable (on) and below detection threshold (off), see e.g. [25], [26]. *Self-organized critical* (SOC) dynamics corresponds to details of regulatory dynamics ensuring (approximate) stability within a fluctuating environment through various mechanisms of adaptation. Finally the property of *differentiability* means that cells of multicellular organisms can differentiate into various cell-types. The differentiated cells possess identical GRN but express distinguishable patterns of regulatory activity. The same GRN therefore can be expressed in different *modes* so that some agents become expressed in one mode but not in another [27].

Recently it has been reported that both regulation of transcription and mRNA decay rates (i.e. the mRNA turnover) are necessary to understand experimentally observed expression values [28]. Moreover it has been demonstrated that decay rates of mRNA are cell-type specific [29]. Analogously for proteins, where the dominant mechanism is the ubiquitin driven proteolysis in the proteasome [30], protein abundance and therefore their degradation has to be tightly controlled [31]. Also the abundance of proteins and whether certain proteins are produced or not is again cell-type specific [32], [33]. This indicates that decay-rates and their control play a crucial role in cell-differentiation. It may be noted that interactions between agents are frequently localized in various cell- *compartments* which usually are not resolved in models of experimental data. Besides active degradation of effector molecules also transport-mechanisms between different cell-compartments, e.g. between the nucleus and the cytosol, can change the concentration of effector molecules (e.g. transcription factor) in the compartment containing their target molecules (e.g. promoter). Thus transport phenomena may also emulate the effect of local production or decay rates.

Variable decay rates however and the property of differentiability are hardly ever considered in GRN models where decay rates of effector molecules (agents) are usually kept constant. Understanding the effects of changes of decay rates of agents therefore is a crucial step towards a deeper understanding of GRN dynamics and the role decay rates play in cell-differentiation. The GRN is the set of all possible interactions of molecular reactions and bindings. The GRN captures all possible features of cells and are responsible for the immense levels of adaptation characteristic to living systems. What happens when different cell-types express the same GRN in alternative ways? At any point in time only small subsets of the GRN are active. Any active subset of the GRN leads to the expression of particular sets of molecules (expression modes). The *active regulatory network* at time 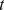 is the regulatory sub-network of the GRN, governing the molecular (auto-catalytic) dynamics of all agents which exist at time 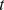. The set of existing effector molecules forms the *active agent set* at time 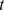. The active network changes over time and typical sequences of active sets represent what we call the *expression modes* of a specific cell-type and its general state. Expression modes themselves can be modified, either locally as a reaction to an external signal, or fundamentally through further cell differentiation. Active sets of molecules are transient and what is observed in experiments is a superposition of subsequent active sets, which we call the *expressed set of agents*. The regulatory interactions between the expressed agents we call the *expressed regulatory network*. To find the property of differentiability in a regulatory network model therefore requires that one network is capable of producing different expression modes while perturbations (external signal) only modify active sets locally and the particular expression mode can be restored.

The six dynamical properties we have listed above have been addressed with a variety of conceptually different models. The essence of all these models is that they try to capture the dynamics induced by positive and negative feed back loops within the GRN. The choice of model depends largely on the type and resolution (coarse graining) of experimental data. At the single cell level cellular activity can be modeled by nonlinear (stochastic) differential equations [34], [35] which can explain homeostasis, periodic and multi-stable behavior. To do so one considers a dynamic variable 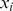 associated with each agent 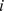. If index 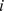 represents a type of effector molecule, then 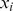 denotes the concentration (abundance) of those molecules. If index 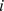 for instance marks the 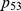 protein 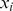 is the 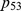-protein concentration (abundance) in the cell. If 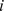 represents a gene, then 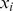 represents the frequency of 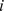 being expressed. The dynamics governed by a GRN is given by a set of coupled nonlinear differential equations
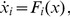
(1)where 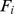 is a (nonlinear) function capturing the GRN. It depends on the vector of concentrations (abundance/activity) of all the possible 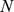 molecular agents in a cell, 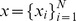. 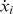 is the time derivative of the concentrations 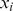. Note that 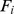 can have stochastic components. Analysis of such systems is often complicated by the interplay between fluctuations and nonlinearities [36].

Differential equation models can be approximated by cellular automata, Boolean or piecewise-linear models. The property of SOC dynamics, or dynamics at the “edge of chaos” [37]–[39], has been studied mainly in the context of cellular automata and Boolean models [40]–[42]. SOC dynamics was also discussed in continuous differential equation based models [43], [44]. Boolean and piecewise-linear models share common origins in the work of Glass and Kauffman, [45], and have extensively been used for modeling and analyzing GRN [46]–[49]. For their superior properties in approximating nonlinear systems (in principle to any suitable precision) piecewise-linear models also are applied in different disciplines, for instance for modeling highly nonlinear electronic circuits [50].

In the context of GRN both boolean and piecewise-linear models usually are used for describing nonlinear dynamics with switch-like regulatory elements frequently observed in biological regulatory processes [51], [52]. Such switches react if the concentration of an agent (the signal) crosses a specific threshold level. To model such switches in regulation networks of 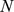 molecular agents with concentrations 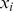 the range (space) of concentrations 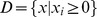 is cut into segments defined by the threshold values where the concentration 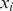 can trigger a regulatory switch. These segments are called *regulatory domains* (e.g. [53]). In each such domain Eq. (1) gets approximated by a linear equation of the form
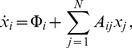
(2)where the 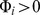 are production rates and 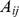 are interaction matrices between agents. If in a regulatory domain 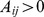, then 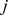 promotes the production of 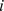. If 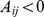, then 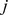 suppresses 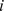. If 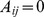 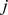 has no influence on 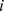. The diagonal elements 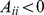 (abbreviated by 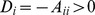) are *decay rates* Such “piece-wise” linear dynamics is nonlinear! On the full range of concentration values 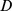 both 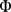 and 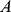 are step-functions depending on all concentrations 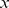 in principle, but being constant in each regulatory domain. In other words, since 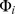 depends only on the regulatory domain it can be decomposed into boolean functions 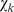 on the regulatory domains. Let 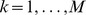 index 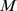 regulatory domains of the system. The function 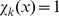 if 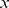 is contained in the 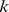
'th regulatory domain and zero otherwise. Then 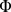 can be written as 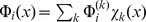 with 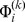 being the value of the production term 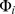 in the 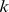
'th regulatory domain. Between regulatory domains the system switches from one linear behavior to another.

As an example for interpreting Eq. (2) consider 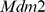 (assign index 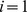) and 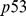 proteins (assign index 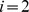). 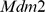 is an enzyme that can act as an E3 ubiquitin ligase on itself and on the tumor suppressor protein 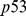 encoded by the 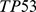 gene. 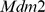 therefore down-regulates itself and the 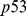 protein via the ubiquitin-proteasome pathway. In the model this corresponds to interaction weights 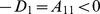 and 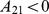. Further 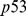 promotes the transcription of 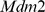, so that 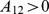, and 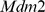 can block the N-terminal transcription-activation domain (TAD) of 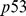 so that 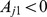 for transcription factors 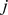 which are activated by 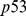-TAD (implying 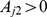). Assuming that 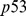-protein does not degrade on its own, i.e. 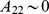, and that both 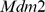 and 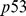 are synthesized at some average rates 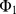 and 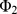, this leads to a linear model of the 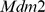-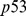 interaction where 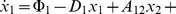 *sum over other influences* and 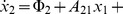 *sum over other influences*.

Given that the interaction matrix 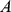 of the regulatory network is invertible (which is almost certainly true for the biologically relevant range of connectivities of GRN) Eq. (2) can be rewritten
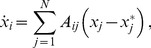
(3)with 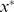 being the solution of the equation 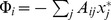. The fixed-point 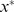 is stable (unstable) and 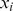 will be attracted (repelled) by 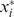. If 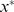 is stable and 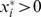 for all 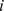 then 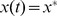 is one (of possibly many) stationary solution of Eq. (2).

Not all models approximating nonlinear differential equation descriptions of GRN are equally suited to capture all GRN properties discussed above simultaneously depending on whether discrete (Boolean, cellular automata) or smooth (differential equation) features dominate the model. However there exists a surprisingly simple class of models which exhibits *all* desired GRN properties.

Here we present such a simple model that captures all of the above dynamical properties. We find that the alternating dynamics plays a key role for the stability of regulatory systems and for the formation of SOC dynamics in particular [43], [44]. Most importantly we are able to show that even unspecific control over decay rates, changing the magnitude of all decay rates simultaneously by a (small) factor, leads to “cell differentiation”, i.e. the same regulatory network enters different expression modes, displaying different sequences of active regulatory networks.

We show that experimental facts, linking variations of decay rates observed between different cell-types of an organism to variations of the abundance of intra-cellular biochemical agents in these cell-types, correspond to (a) differences in the *expressed* genetic regulatory network, and (b) these differences can be controlled via decay rates of intracellular agents. In other words typical expression modes (cyclical sequences of successive active sub-networks of the GRN) can be altered and switched by controlling decay rates.

### The model

Setting 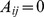 in Eq. (2), except for the (usually) fixed decay rates 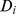, leads to a set of equations 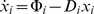. Since 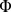 and 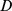 may depend on the regulatory domain this corresponds exactly to the class of Glass-Kauffman piece-wise linear models, [45], [53]. In Glass-Kauffman systems, [45], concentrations 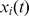 usually remain positive for all times 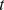, given positive initial conditions 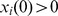 and 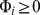 for all 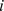 since concentrations 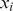 can at best decay exponentially with time (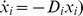. This makes it impossible to produce alternating activity of agents. For 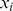, in a Glass-Kauffman system, to become zero within a finite time, production rates - which are non-negative by definition - would have to become negative.

Equation (2) generalizes this class of models to systems allowing to explicitly model linear regulatory interactions 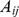 between agents within each regulatory domain. Suppose 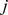 suppresses 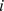 (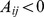) then 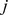 can in principle down-regulate 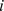 in a finite time (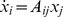) and positivity of solutions of Eq. (2) is no longer guaranteed. Positivity (non-negativity) of solutions needs to be ensured as a constraint on the piece-wise linear dynamics
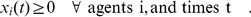
(4)This constraint alters the linear dynamics of Eq. (2) in the following way. Whenever a concentration 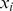 becomes zero at time 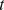 then 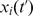 remains zero for 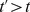 for as long as 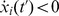, according to Eq. (2). If 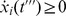 for 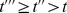 then 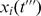 is no longer subject to the positivity constraint and continues to evolve according to Eq. (2) again. Agent 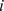 is said to be *active* at time 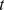, if 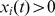 and *inactive*, if 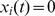. To simplify the discussion in the following we only consider systems with a single regulatory domain - such that all nonlinear behavior of the dynamics is solely due to the positivity-constraint.

The positivity constraint Eq. (4) implies the following consequences. At any point in time there will be a sub-set of agents with non-vanishing concentrations which we call the *active set* of agents. The remaining agents have zero concentration, and therefore do not actively influence the concentrations of any of the non-vanishing agents. There exist 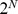 different active sets, i.e. 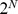 combinations in which 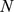 agents can be active or inactive. Each active set can be uniquely identified by an index 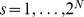 (e.g. 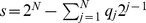 with 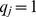 if the 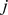
'th agent is active and zero otherwise). In the course of time 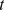 some agents will vanish while others re-appear, so that one effectively observes a sequence of sets of active agents
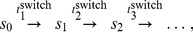
(5)

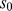
 being the initial active set. The active set 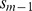 switches to active set 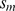 at time 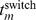. In each time interval 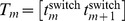 of duration 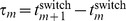 it is thus possible to only consider the regulatory sub-network acting on the set of active agents 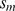. This sub-network is described by the part of the full interaction matrix 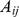, where 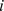 and 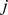 are restricted to the set of active agents 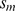. These sub-matrices we call *active networks* and denote them by 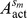. The concentration vector of active agents we call 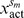. Active agents also “feel” a modified *effective* fixed point 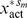, such that finally for 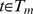 the concentrations of the active agents follow a linear equation
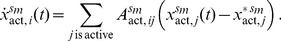
(6)We refer to such systems as *sequentially linear* systems. The attractiveness of this description arises through the fact that it becomes possible to understand the dynamics by considering the sequences of active networks
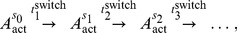
(7)which allows to analyze dynamical properties in terms of eigenvalues and eigenvectors of the active sub-matrices 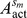 (see materials and methods). This model can be shown to be mathematically equivalent to [43], [44].

### Cell differentiation in the sequentially linear dynamics

The dynamics of nonlinear systems in general and sequentially linear system in particular converges to different *attractors* of the dynamics (fixed points, limit cycles). Which attractor is “found” depends on the initial condition. Sequentially linear systems can possess multiple distinct limit cycles and fixed points. Perturbations (or different initial conditions) may push a system from one to another attractor. The question of how many different attractors a sequentially linear system possesses goes beyond the scope of this paper and will be discussed elsewhere.

In the picture of sequentially linear dynamics it becomes possible to identify operational modes of a cell as a particular sequence of active networks. Cell types in ordinary operational modes may be classified by specific sequences of active networks. Two distinct possibilities for such sequences exist. One possibility is that, after some initial switching events, a system ends up in a stationary state associated with a particular active network of the system (see materials and methods). The other possibility is that a system converges to a periodic dynamics with an associated periodic sequence of active networks.

As a hypothetical example a liver cell under typical conditions might be characterized by a *periodic* sequence 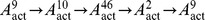, whereas an endothelial cell is given by 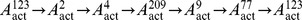. Note that all types share the same full regulatory network 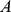. This separates timescales of the dynamics: on the fast timescale the dynamics is continuous and characterized by linear changes of the concentrations 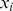. On the slower time-scale the dynamics is characterized by discrete changes of active sets. The change from one sequence of active sets to another can be interpreted as the expression modes of different cell-types (cell differentiation) and we show that changes in decay rates of molecular species trigger switches between expression modes.

### Example

**Figure 1:**
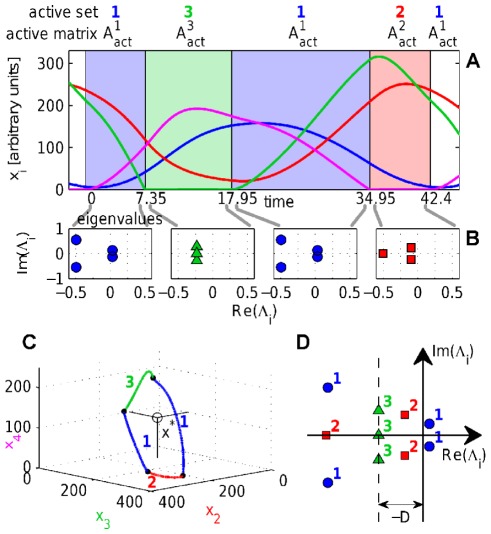
Periodic dynamics and active sets. Sequentially linear system with decay rate 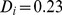 and the fixed point 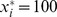 for all agents 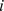 simulated with time-increment 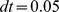. Periodic time-series organized into a sequence of four domains with three different active sets. For each time-domain the associated spectrum of eigenvalues for the active sets is shown in (b). In (c) a 3 d Poincare map of the limit cycle is plotted together with the projection of 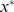 in the center. The domains are marked with bold numbers and switching events with dots. (d) The eigenvalue spectra of the different subsystems are plotted in the imaginary plane. The shift of the spectrum along the real axis depending on the decay rate 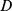 is indicated.

As an example for sequentially linear dynamics we consider a system with 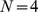 molecular agents, 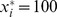, 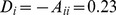 for all agents 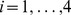, and a regulatory network given by
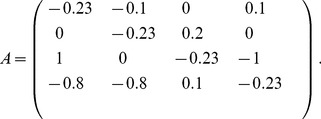
(8)The dynamics of this system (over one period) is shown in Fig. 1 a. The property describing the stability of an active set 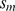 is the maximal real part of the eigenvalues 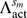 of the active matrix 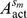 denoted 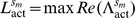. The number 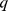 denotes the number of time-domains in a periodic sequence of active networks, i.e. the number of switching events per period, and 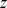 is the number of different sub-networks that are activated in a sequence (see also materials and methods). In this example there are four time-domains (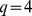) associated with three different active sets (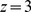) which are periodically repeated. The sequence starts in time-domain 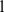 with active set 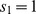 (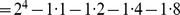) with maximum real eigenvalue 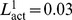. Positive 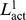 means that the fixed point of the active set is unstable and the associated leading eigenvalue implies that the concentration of one agent (green) is decaying to zero. The positivity condition deactivates this agent as its concentration becomes zero and the system enters time-domain 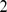 as the active set switches to 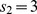 (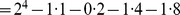) with 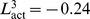. Negative 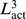 means that the fixed point 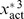 is stable and 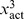 tries to approach 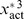. This leads to the deactivated agent (green) becoming produced again and the system switches back to 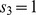 entering the third time-domain. In time-domain 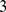 the initial conditions differs from the one in time-domain 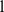 and a different node (magenta) gets deactivated. The system switches to 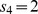 (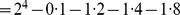) with 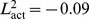 at the beginning of the fourth time-domain. This means 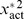 is a stable fixed-point and the inactive node (magenta) eventually gets produced again as the system switches back to the beginning (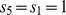) and enters the next period. The system is thus precisely characterized by the sequence 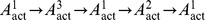. The eigenvalue spectra of the sub-matrices 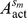 associated with subsequent time-domains 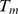 are shown in Fig. 1 b. Fig. 1 c shows a projection of the trajectory into a three dimensional Poincare map. Fig. 1 d shows the eigenvalue spectra of the different active sub-systems of the dynamics.

Some details of the dynamics, like the existence of multiple stable fixed-points, the periodicity of bounded attractors and temporal self-organization, can be mathematically fully understood. In [43], [44] it was already shown mathematically that sequentially linear models exhibit *homeostasis*, and *multi-stability*. This has been demonstrated for a wide range of system size 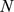, and a number of interactions (connectivity) and fixed decay rates. *Periodic dynamics*, and *self-organized critical* dynamics have been noted in [43], [44] but were not clarified and require further explanation which is given in detail in *materials and methods* , where also a simple temporal balance condition is described and derived.

The temporal balance condition states that the time-average over the real parts of the leading eigenvalues 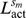 of the matrices 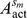 in a sequence of active networks approximate the Lyapunov exponent 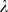. The Lyapunov exponent 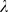 measures the overall stability of a system (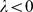 stable, 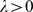 instable, 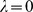 critical) and for sequences following a periodic attractor 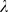 can be shown to be exactly zero. Inserting the values for 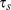 and 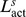 from table 1 into the balance condition, Eq. (11) gives the value 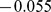 as an approximation of 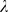 (which has an exact value of zero). Although the balance equation gives only a crude approximation of the Lyapunov exponent it allows to understand why the example-system spends more time in the weakly instable time-domain 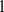 and 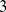, than in the stable time-domains 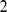 and 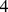 which is obviously true from Fig. 1. Strong convergence needs less time to compensate for weak divergence.

**Table 1:** Domain properties.

time-domain	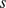	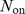	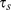	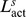	stability
1	1	4	7.35	0.033	unstable
2	3	3	10.6	−0.24	stable
3	1	4	17.0	0.033	unstable
4	2	3	7.45	−0.094	stable

Some characteristics of the four node system shown in Fig. 1 are listed, including the index of the time domain, the index of the sub-system 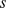, the number of active nodes 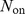, the time the system spends in the 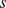
'th sub-system, the real-part of the leading eigenvalue of 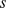, and whether sub-system 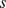 is stable or not.

Temporal balance is a consequence of the mechanism of self-organization that fine-tunes switching times such that stable parts of the dynamics compensate instable parts of the dynamics exactly. This mechanism can be understood in the following way. Sequentially linear systems try to converge to a fixed point. If it is reached the system becomes static. The fixed point might not be *accessible* however, meaning that the trajectory on the way toward the fixed point hits a boundary (Fig. 1 c) causing a switching event which changes the dynamics so that the system now is attracted by a different effective fixed point, which it tries to reach. If the system does not converge to an accessible fixed point it is either unstable and some concentrations 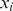 diverge, or the system circles through some of the 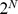 possible active sets and converges onto an effective attractor - characterized in the sequence of active networks. In the later case small perturbations of 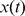 on the attractor will vanish with time. This allows to show that bounded dynamics that does not converge to a fixed-point has to be periodic (materials and methods). Switching times are not static but react to perturbations of concentrations 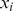. Perturbations shift the occurrence of switching times proportional to the magnitude of the perturbation. This has the effect that switching events act like sliding “focal planes” allowing the perturbed dynamics to “refocus” onto the periodic attractor. While the perturbed dynamics returns to the attractor switching times cumulate small time-shifts resulting in a phase-shift of the periodic dynamics. A perturbation is remembered as a phase-shift of the periodic dynamics which neither grows exponentially nor dies out. The Lyapunov exponent therefore is zero and the systems self-organizes to the “edge of chaos” by adaptation of switching times. Stable adaptive dynamics is a result of this “temporal self-organization”.

## Results

**Figure 2:**
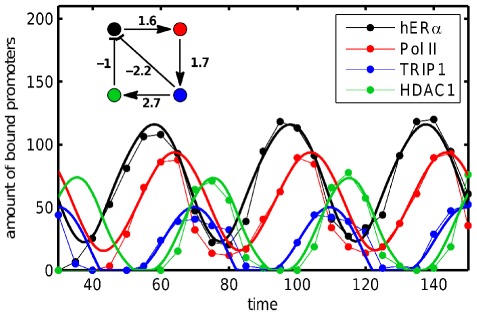
Adequacy of sequentially linear systems. Time series of periodic binding of four proteins to the pS2 promoter after addition of estradiol - experimental data has been extracted from [54], where a negative feedback-loop was proposed to explain the dynamics. Experimental data due to [25] and [26] (dotted lines) is compared with a simulation of a SL system, based on the network shown in the inset, with uniform decay rates 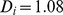 for all agents and fixed point concentrations 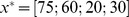. Correlation coefficients for simulated and measured time-series are 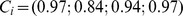 for time larger 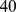 and agents 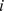 in order of the legend. The model simulation uses zero concentrations for all agents as initial condition and a time increment 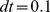. For matching the simulation with experiment time in the model is shifted by 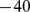.

We first show that the model is able to explain actual empirical data, including alternating dynamics. Figure 2 shows data of molecular concentrations 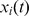 (hER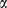 (black), Pol II (red), TRIP1 (blue), HDAC1 (green)) over three periods of about 40 minutes time. These four agents are all part of the human estrogen nuclear receptor dynamics. The source of the Data is Metivier et. al. [25]. Data points were taken from Pigolotti et al. [54] and the actual values of the matrix elements
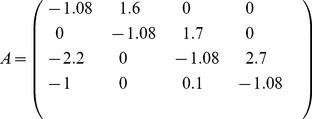
(9)are bests fits with identical decay rates for optimal explanation of the data. The TRIP1 data (blue) shows *alternating activity* which is reproduced perfectly by our sequential linear model.

### Decay rates and expression modes

In the following we show how the change of decay rates induces changes from one cell-type to another. In particular we show how changes of the overall strength of the decay rates results in differentiated dynamics, i.e. in distinct sequences of active expressed networks. This allows to understand recent experimental observations which indicate correlations between cell-type, expressed sets of agents, and decay-rates [27]–[29], [31]–[33].

**Figure 3:**
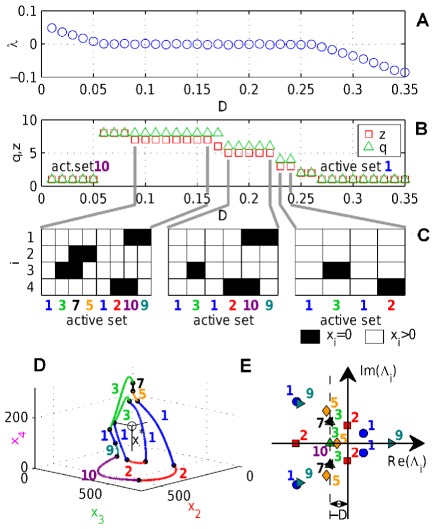
The edge of chaos. The Lyapunov exponent 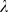 of the four node system, Eq. (8), is shown in (a) as a function of the decay rate 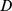, which exhibits a “plateau” with 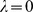 in the range 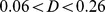. In (b) the length 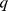 of the periodic sequence of domains is plotted in green triangles and the number of different active sets 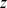 as red squares. In (c) the sequences of active sets are shown for decay rates 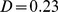, 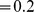 and 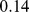. The limit circles for decay rates 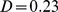 (short sequence) and 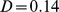 (long sequence) are visualized in (d) in a Poincare map using three out of four phase-space dimensions. With decreasing 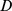 the radius of the limit circle becomes wider and additional sets (marked with colors) become active. In (e) the spectra of eigenvalues are shown for all the appearing active sets with 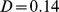.

For a fixed interaction network temporal self-organization can be maintained for a wide range of decay rates 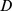. We show this in the same 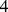-node system considered in Fig. 1 by only varying the decay rate 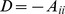 from Eq. (8). Figure 3 a shows the Lyapunov exponent 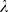 as a function of 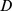. A plateau, where 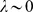, is clearly visible. If the decay rate is larger than a critical value 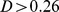, the Lyapunov exponent becomes negative (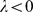) and the system stable. If the decay rate is smaller than a critical value of 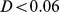, temporal balance can not be achieved any more, refocusing breaks down, and the system becomes chaotic and trajectories diverge exponentially with 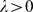. In Fig. 3 b the length of the periodic sequences 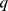 (green triangles), which is the number of time-domains in a sequence, and the number 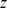 of different active sets activated in this sequence (red squares) is depicted. Figure 3 b also shows that at several critical values of 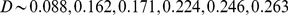 in the plateau region the sequences of active regulatory sub-networks changes when temporal balance can no longer be established merely by adapting the switching times of a sequence. Sequences do not usually change completely at critical values of 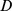 and are only expanded by additional active subsets. This can be seen clearly in the 3D Poincare map of the dynamics Fig. 3 d, where the sequence of subsystems 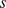 given by 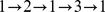 (for 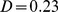) gets expanded to the sequence 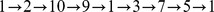 (for 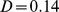).

The mathematical reason why such critical decay rates exist is that changes of 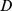 shift the eigenvalue spectra of the active interaction matrix 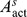, shown in Fig. 3 e, along the real axis. The real part of the leading eigenvalues, 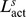, is becoming smaller (larger) than zero and 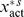 becomes an attractor (repellor) of 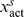. The stable fixed point then either is accessible and the dynamic changes from periodic to stationary or inaccessible and the dynamic changes qualitatively but remains periodic. Which agents become active in a given active set 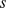 is depicted in Fig. 3 b for three different sequences of active sets associated with three different ranges of the decay rate 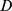 indicated by gray lines. If node 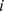 is active in active set 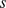 then the associated field is white and black otherwise.

**Figure 4:**
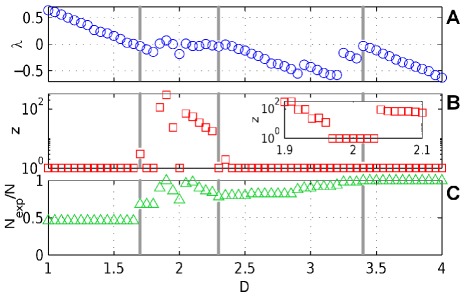
Degradation rates and active networks. Example of a SL system with 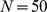 and 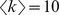 and identical initial conditions for all values of 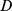 expressing different portions of the regulatory networks. (a) The Lyapunov exponent, (b) the number of active sets 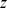 in a period (if 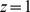 then the sequence is not periodic but a steady state!), and (c) the fraction of expressed nodes are plotted as functions of the uniform decay rates 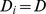. For 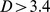 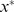 is stable. In the range 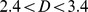 the 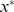 has become unstable but the plateau (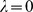) can not form since the dynamic finds active sets 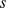 with stable and accessible 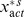. The inset in (b) shows that in the plateau region a small window, 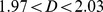, exists where again an active set 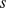 contains an accessible 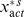 attracting the dynamics. In the range 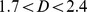 the plateau forms and dynamics gets periodic. For 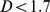 the system gets unstable.

The number of *expressed* agents 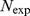 is the number of agents that are active at least once during a period of the dynamics. To demonstrate that not only the periodic activation of agents depends on 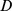 but also the number of expressed nodes 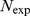 itself, we consider a larger sequentially linear system with 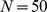 agents. The interaction matrix of the system is a random matrix with average connectivity 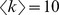, meaning for each node 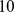 interactions with other agents have been randomly chosen with equal probability. Each non-zero entry, describing such an interaction, is drawn from a normal distribution with mean zero and a standard deviation of 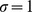. This means that the interaction strength is of magnitude 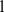 on average and has positive or negative sign with equal probability. In Fig. 4 a the Lyapunov exponent 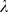, in Fig. 4 b the number 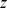 of sets that become active during a cycle and in Fig. 4 c the fraction of expressed agents 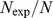 is plotted as a function of 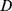. For large decay rates (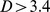) the system is stable and 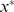 is a fixed-point of the dynamics. As 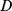 decreases 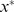 becomes unstable for 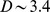. However for 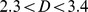 the system ends up in some stable accessible fixed point 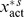 so that 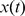 approaches a stationary state and 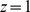. In this range 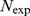 increases with 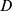. The 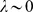 plateau with stable self-organized critical dynamics (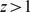) only emerges in the range 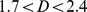 where number of active sets 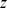 and expressed network size 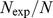 vary strongly. 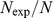 varies between 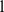 and 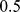 which means that changes of the decay rate can induce changes of the size of the expressed network comparable to the magnitude of the full interaction network. A small window of stability exists for 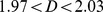 (see inset).

The strong dependence of 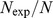 on the decay-rate 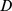 (up to 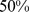 of the total regulatory network) demonstrates clearly that decay-rates alone massively influence sequences of active systems without changing the interaction strength between agents in the regulatory network at all. Moreover, decay rates can also cause switches between fixed-point dynamics and periodic dynamics. While fixed points favor larger decay-rates (in the example 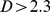) there can also exist fixed points for smaller decay rates (window of stability 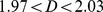) where systems favor periodic dynamics.

## Discussion

We presented a model which de-composes the dynamics of molecular concentrations – governed by the full molecular regulatory networks – into a temporal sequence of active sub-networks. This novel type of model allows not only to reduce the vast complexity of the full regulatory network into sub-networks of manageable size but further to approximate the complicated dynamics by linear methods. The intrinsic nonlinearities in the system which lead to alternating dynamics in concentrations (as found in countless experiments) are absorbed into switching events, where the dynamics of one linear system switches to another one. In this view different cell types correspond to different sequences of active sub-networks over time.

These sequentially linear models allow not only for the first time to describe all the relevant dynamical features of the GNR (homeostasis, multi-stability, periodic dynamics, alternating activity, differentiability, and self-organized criticality), but also offers the handle to understand the role of molecular decay rates. The fact that sequentially linear dynamics properly models homeostasis, multi-stability and periodic behavior was shown in [43], [44]. Here we have shown how self-organized criticality (Lyapunov exponent self-regulates to zero) arises as a consequence of temporal balance of switching events. This requires agents to show alternating activity (being repeatedly on and off), which is a natural property by construction of sequentially linear models, and which has posed an unresolved problem of previous models such as the Glass-Kauffman [45] model and its many variants. The mechanism behind self-organized criticality is based on adaptive switching times which effectively lead to refocusing of perturbed dynamics onto the attractor of sequences of active sub-networks. Such a temporal self-organization causes long time memory of perturbations in terms of phase-shifts of the otherwise unchanged periodic dynamics, causing the Lyapunov exponent to become zero. In other words slight perturbations, e.g. noise, only cause time-shifts of the sequence of regulatory reactions but do not change the underlying sequence. Perturbations are “remembered” by the system by non vanishing phase-shifts and the dynamics gets “refocused” onto the periodic attractor merely accumulating a time-shift. This has the consequence that the Lyapunov exponent is zero and the system self-organizes its criticality by adapting switching-times. Practically this means that a system balances the time it spends in its active sub networks with stable and unstable dynamics (temporal balance).

Applying the sequentially linear model to the problem of cell-differentiation we demonstrate that different levels of decay rates are one to one related with transitions from one active sub-network sequence (cell type) to another. This might be a key ingredient to understand a series of recent experimental facts reported on the role of decay-rate regulation systems and the role of noise in cell differentiation [27]–[29], [31]–[33]. We found that varying the decay rates only, while keeping the complete regulatory network fixed over time, substantially modifies the temporal organization of regulatory events. In particular the decay rate controls the number of expressed agents, the sequence of active sub-networks, and sometimes even the type of solution (stationary, periodic). The changes occur at critical levels of decay rates and changes can be drastic. For example we find situations where a 5% variation of the decay rate causes an approximate doubling of the number of expressed agents. In [55] it is argued that (in the regulatory core and bottom layers of a regulatory hierarchy) transcription factors abundance may be kept low by tightly controlled degradation effectively acting as a noise filter enhancing fidelity in gene expression and adaptability to changing environments. This makes sense from a theoretical point of view first of all since fluctuations of agents with low abundance are more likely to trigger switching events in the regulatory dynamics leading to distinct global responses of the regulatory network. Secondly, degradation and re-synthesis of agents with high regulatory activity consumes energy so that a low abundance of regulatory important agents is consistent with cells evolving under a constraint of energy-efficiency. Similarly, based on measurements of mRNA and protein decay-rates [56] argue that, while abundance of mRNA and protein over and all may be controlled by transcription rates rather than decay-rates, proteins with short half-lives mainly have regulatory function (Chromatin organization and modification, cell cycle, mitosis and cell proliferation, transcription, homeostasis, proteolysis, …). This demonstrates that different expression modes, which distinguish different cell-types from each other, can be very efficiently obtained by controlling decay rates (either via proteolysis or alternatively via transport mechanisms controlling the local abundance of effector molecules in compartments containing associated targets) of agents without fundamentally altering any interactions between agents in the regulatory network, which would be more costly in an evolutionary sense. These findings highlight the importance of intracellular decay rate control mechanisms and the role of noise in cell differentiation.

## Materials and Methods

### Eigenvalues

The eigenvalues 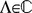 and eigenvectors 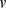 of a matrix 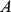 are defined as solutions of the matrix equation 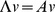. The solution of a linear differential equation 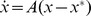 is of the form 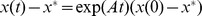. For large times the 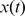 will therefore point into the direction of the eigenvector 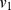 with the eigenvalue 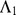 with the largest real part and 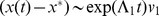 as 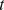 gets large. If the largest real part of 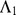 is larger (smaller) than zero 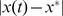 will grow (decay) exponentially and 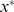 is an unstable (stable) fixed point of the differential equation.

### Fixed points and attractors

Let 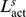 be the maximal real part of the leading eigenvalue of the active interaction matrix 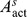 associated with the active subset 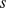. The effective fixed point 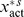 is *stable* and perturbations of concentrations vanish if 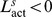. The fixed point is *accessible* if 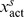 approaching 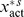 does not cause a switching event and *inaccessible* otherwise. Stationary solutions of a sequentially linear system therefore require fixed points that are both stable and accessible.

Stable and accessible fixed points can be fully understood. Suppose agents 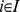 are active and agents 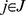 are inactive then Eq. (3) can be rewritten into two parts
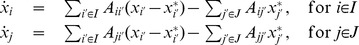
(10)where the first part describes the dynamics of the active agents while the second part is the part of the linear dynamics superseded by the positivity constraint. Symbolically we can write 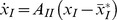 with 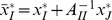 and 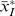 is the modified fixed point. For 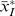 to be a accessible one requires that 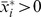 for all 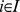. For 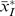 to be stable one requires two things. (i) The real part of the leading eigenvalue of 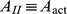 needs to be smaller than zero. (ii) 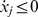 for all 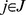. If the second condition is violated for some 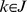 then 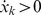 so that in the next time step a switching event occurs since 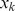 becomes larger than zero and is no longer controlled by the positivity constraint.

### Periodicity of attractors and self-organized criticality

**Figure 5:**
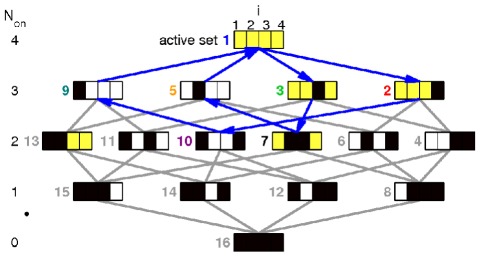
Tree of active sets. Tree of all existing active sets 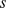 for system shown in article Fig. (3). In set 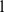 all 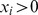, yellow background stand for complex leading eigenvalues of the active interaction matrix. Black indicates that the agent associated with that index is not active. The gray lines indicate to all possible switching events where the number of active agents 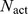 changes 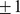. Blue arrows mark the observed sequence of the dynamics for the examples Eq. (8) with 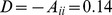.

We have seen that attractors either are fixed points or periodic. The longer periodic sequence of Fig. 3 d is also shown in the space of all possible active sets in Fig. 5. But can one understand why bounded dynamics is periodic rather than chaotic? Suppose a bounded attractor exists for a sequentially linear system with 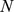 agents 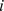. The perturbation 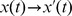 at time 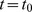 also effects later switching times of agents 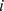, i.e. 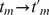 such that 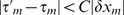 for some constant 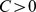, where 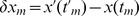. Since 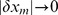 sufficiently fast as 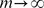 (there exists an attractor) the cumulated time shift 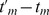 of switching times remains finite for all times. This shows that the perturbed 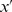 behaves (after some time) just like the unperturbed 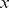 only shifted in time. Perturbation neither vanishes nor grow exponentially, and the Lyapunov exponent can only be zero (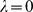). Moreover, since the number of active sets is finite (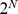) and the dynamics is bounded the concentrations have to return to values on the attractor with arbitrary precision within some finite return-time. The remaining concentration difference can be seen as a perturbation so that the attractor can only be a periodic cycle. The time-shift produces a phase-shift of the periodic dynamics.

### Stability: the maximum Lyaponov exponent

While eigenvalues tell us something about the stability of a fixed point the Lyaponov exponent 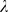 tells something about the stability of the dynamics 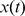 itself. The Lyapunov exponent 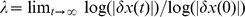 measures how a small perturbation 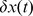 grows with time. If 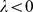 the perturbation vanishes exponentially with time or grows exponentially if 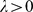. System with 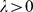 are chaotic (in-stable dynamics extremely sensitive to noise or perturbations) while 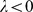 indicates stable dynamics insensitive to perturbations and noise. Systems with 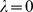 are special as their dynamics is sensitive to noise and perturbations without “overreacting” like chaotic systems. These systems at the “edge of chaos” adapt to fluctuations but remain close to their unperturbed dynamics.

### Temporal self-organization of switching events

Here we derive a simple approximation of the Lyapunov exponent of sequentially linear dynamics which explains temporal self-organization quantitatively. This is necessary for understanding why switching in general happens between active networks with stable and unstable dynamics and not from one stable stable (unstable) to another stable (unstable) active network.

Qualitative analysis of bounded attractors of sequentially linear dynamics has shown that the attractor is periodic and the Lyapunov exponent 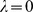. Characteristic information on the dynamics gets encoded by periodic sequences 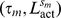, 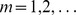 with a period of some length 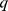 such that 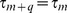 and 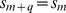 (for large enough 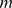) as in the example shown in Fig. (1) in the main article. If the dynamics of the system would remain in an active network 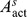 the Lyapunov exponent would be identical with the largest real part 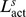 of the eigenvalues of 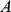. However, note that convergence of 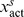 to 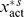 (if fixed point is stable) or into the direction of the leading (possibly complex) eigenvector (if fixed point is stable) remains incomplete, since convergence is always interrupted by a switching event. The Lyapunov exponent 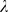 of the sequentially linear system therefore is well approximated by the time average over 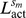, i.e.
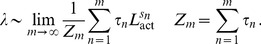
(11)Since the dynamics is periodic the time average only needs to be taken over one period and since 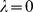 one gets 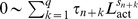 for 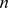 large enough. The “refocusing” mechanism discussed above qualitatively therefore is also “balancing” the times 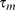 specific active sets 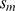 remain active by fine tuning switching times, such that contributions from time-domains with stable (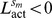) and unstable dynamics (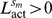) compensate each other. This also is supported by the fact that simulations with finite time increment regularly produce chaotic dynamics with small but positive Lyapunov exponents since switching times can only be tuned to the accuracy of the time increment. However 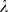 approaches zero consistently as the time increment is made smaller and orbits become periodic again. *Temporal balance* and *refocusing* are two aspects of the temporal self-organizing principle manipulating switching times.
